# Computational biologists: moving to the driver's seat

**DOI:** 10.1186/s13059-017-1357-1

**Published:** 2017-11-23

**Authors:** Itai Yanai, Eva Chmielnicki

**Affiliations:** Institute for Computational Medicine, NYU School of Medicine, New York, NY 10016 USA

## Abstract

The recent shift of computational biologists from bioinformatics service providers to leaders of cutting-edge programs highlights the accompanying cultural and conceptual changes that should be implemented by funding bodies and academic institutions.

## Introduction

Computational approaches began to revolutionize the life sciences a generation ago, when DNA sequences became more widely available [1]. Sequence analysis methods—most notably BLAST—were crucial in uncovering the molecular basis for deep homology across distant organisms, as well as rampant, and previously unappreciated, horizontal transfer across organisms [2]. Currently, the volume of data generated and processed in the course of modern scientific research is growing exponentially [3]. Large databases are proliferating with myriad types of biological data, such as the Genomic Data Commons platform, which provides researchers access to genomic and clinical data from patients with cancer [4]. More recently, insights into cancer biology have emerged from single-cell surveys of tumors [5]. Such computational analyses have altered the current thinking about tumor architecture, and the tools used—most notably sequence and gene expression analysis—have permeated the life sciences.

As a consequence of these changes, computational biologists must invent analytical algorithms and deploy them to make scientific breakthroughs. Here, we describe five specific areas in which the role of computational biologists has shifted over the past 20 years (Table 1). Within each of these areas, we present examples of what has changed, and how computational biologists can work with more traditional life scientists to increase the probability of groundbreaking discoveries across biomedicine.

**Table 1:** Shifting roles of computational biologists

	Past	Current
Role in research	Supportive	Driver of research
A feeling for the biology	Computer science-centered	Biology- and computer science-centered
Environment	Isolated	Integrated
Data generation	Constrained	Resourceful
Data exploration	Largely limited to hypothesis testing	Both exploratory and hypothesis testing

## Role in research

Computational biology originated as a tool rather than as its own discipline as it did not embody a set of core questions. Thus, bioinformaticians have historically had a supportive role in research programs led by other scientists who decided on the tractability and value of the scientific questions to be pursued. Bioinformatics, therefore, belonged more as a subheading in the Methods section than as a title of a manuscript. Yet those who are able to make sense of the richness of data in the modern life sciences have now been put in the driver’s seat. As a result, computational biologists are now often principal investigators on grants, rather than co-investigators, and they are also last authors on groundbreaking publications.

This change occurred because many fields now present opportunities for computational explorations of patterns and integration across diverse and rich data sets. An example is the development of RNA-sequencing (RNA-seq). Bioinformaticians were involved in setting up the statistical framework for analyzing the data and producing a gene expression matrix [6]. However, beyond the technical details of the method, computational biologists have been instrumental in exploring single-cell RNA-seq data sets and revealing important new insights, as evidenced by the proliferation of single-cell RNA-seq papers in top journals and at conferences worldwide. The number of single-cell RNA-seq papers in the literature has increased fivefold in the past 5 years, with many of these papers being led or co-led by computational biologists.

Much of the power of computational biology follows from its use in collaboration with experts in other fields. How then are computational biologists to collaborate as equals with more experimental biologists? We have found that a key aspect in starting a collaboration is finding a “win-win” situation to working together. Often this comes from establishing not one but two parallel projects; with each lab leading one project. Thus, neither lab is second to the other in the collaboration and both projects gain from the interaction.

## A feeling for the biology

PhD training programs now enable the development of leading scientists who are not only interested in algorithm creation and data analysis but who are also keenly aware of the most pressing questions in biomedical research. With the “feeling for the biology” gained by such training comes a drive to address the central problems of the field in question. For example, to have a feeling for cancer one must understand that cancer has a somatic evolution, and to have a feeling for a host–pathogen interaction one must understand that the interaction unfolds over time. Without these insights, an analysis of the data cannot be connected to the biology in a way that elicits discoveries. Similar to the experimental biologist, in order for the computational biologist to make the most groundbreaking discoveries in a particular field, they must be tormented by the most pressing biological problems in that field of study.

In order for computational biologists who are currently in training to obtain such a feel for the biology, they must have ample opportunities to discuss key biological concepts with life scientists who focus on the same field, such as cancer or immunology. Moreover, future training programs will need to teach computational biology approaches to all students, not only those with a more computational biology bent, such that computational approaches increasingly become part of the general language of science.

## Environment

By virtue of the classical university departmental structure, when computational researchers began launching independent labs they would often be surrounded by traditional life science researchers who could not speak the language of quantitative biology. This isolated structure did not allow for computational biologists to learn from each other or provide a critical mass for computational thinking. Examples of an analogous shift can be seen when looking at stem cell researchers, who were previously isolated within separate departments: a skin stem cell researcher would have been in a dermatology department, and a neural stem cell researcher would have been in a neuroscience department. But with the launch of many stem cell institutes in the 1990s, these investigators had more opportunities to stimulate and inspire each other.

The specific topics of a microbiome computational biologist and a cancer computational biologist, for example, will vary, but by increasing their interactions they will inspire and cross-fertilize each other’s fields. This is because of a common set of tools from which all computational biologists draw, such as modeling methods for systems-level analyses, statistical methods for assaying significance of specific hypotheses, and visualization for reducing the high-dimensionality of data sets.

## Data generation

In the past, by virtue of their focus on computational approaches and lack of expertise at the bench, computational biologists would focus on data sets that were available in the public domain or from collaborators. This constrained computational researchers from creating their own data sets, and from being able to validate any hypotheses arising from their computational approaches.

However, computational biology is now more integrative both in how it generates and in how it interprets data. From this perspective, the typical computational biology lab may look very similar to a molecular biology lab in that its members have both bench space and private computer workspace. Moreover, novel data sets generated in the lab are often best studied with respect to other data sets. This requires the computational biologist to be a “Renaissance scientist”, with multiple experimental and computational tools, as well as to have the ability to take advantage of theoretical models and data described in the literature.

The quality of the findings derived from a complex data set likely requires both a deep understanding of biological questions and algorithm innovation. Therefore, we may be witnessing a change in what “ownership” means for data. A de facto rule in the culture of life sciences has been that data is “owned” by those who generated it. Thus, an RNA-seq data set is owned by the person who generated it using commonly available methods and only requiring a short period of work. The data set may have been subsequently intensely analyzed for a considerably longer period by another person, who used specialized computational tools and biological insight to make a discovery. However, as we have witnessed many times, the person who generated the data has an inherent advantage in becoming the first author on the subsequent paper. This often unfair assignment of credit is now changing, however, as the role of computational biologists is gaining recognition. Funding bodies and institutional tenure committees should also create new credit structures to ensure that those who make important discoveries are promoted and funded regardless of whether they generated the data themselves.

## Data exploration

The ability to generate and test a hypothesis has always been the cornerstone of science. Bioinformaticians have classically used complex statistical methods to contribute to the experimental design of a study and to data analysis for hypothesis testing. However, hypotheses were often generated by the biological experts. Now, with the emergence of large data sets across biomedicine, computational biologists can contribute not only to testing hypotheses, but also to exploring the data in such a way as to generate novel, unexpected, groundbreaking hypotheses that can subsequently be tested and validated in independent data sets, as well as at the bench. Indeed, the recognition that crucial hypotheses arise from data-driven “fishing” expeditions must be communicated to funding bodies and other scientific institutions.

A current stumbling block with computational biology, which we hope to see overcome soon, is the validation of hypotheses inferred from complex data sets. Computational biologists tend to lack extensive validations in their papers, which may indicate a more theoretical bent or a lack of expertise in the required experimental methods. Efforts by computational biologists, however, to improve this aspect of their science are likely to improve their grant applications and to increase their publications in top journals.

## Concluding remarks

In spite of the transformation that we describe here in how computational biologists now contribute to the scientific enterprise, one aspect that has not changed is the need for computational biologists to collaborate closely with experimentalists and clinicians across the biomedical spectrum. The testing of a hypothesis generated by the analysis of a data set needs to be validated in complex experimental systems, such as mice and organoids, or by using interventional in vivo approaches that require a lifetime of expertise in a particular method of drug administration. In addition, the initial hypothesis may need to be validated by collecting new data from an independent human population. This necessitates close interaction with patients, to whom the computational biologist may not have ready access.

The importance of collaborative relationships between wet and dry researchers blurs the lines that define research, and is also reflected in recent changes in how research is funded and organized at the institutional and governmental levels. For example, large collaborative federal grant funding requires clinicians, experimentalists, and computational biologists to come together to interact with data and ideas in ways that are not possible in grants that are awarded to a single principal investigator. Many institutions around the world have launched computational centers and departments, particularly in the field of cancer, and have designed cutting-edge lab spaces where computational and experimental researchers work closely together in collaborative groupings, with many opportunities for frequent daily interactions.

World-class computational biologists must innovate in both the computational and the biological realms so that the secrets hidden in biomedical data can be unlocked. Some cultural changes by funding bodies and academic institutions may be necessary, but when these parameters are met, computational biologists will be able to make the most groundbreaking discoveries.
